# Alzheimer’s disease patient iPSC‐derived neuron‐based high‐throughput screening identifies gut microbial metabolites involved in reduced AD pathology.

**DOI:** 10.1002/alz70859_103052

**Published:** 2025-12-25

**Authors:** Dhruv Gohel

**Affiliations:** ^1^ Cleveland Clinic, Cleveland, OH USA

## Abstract

**Background:**

The gut‐brain axis represents a bidirectional communication network between the enteric and central nervous systems. Recent investigations have demonstrated that gut microbiota modulate brain function and behavior through the production of a diverse array of metabolites and neurochemicals. Understanding the specific gut‐derived microbial metabolites that influence neurodegenerative processes, such as tauopathy, may provide novel therapeutic strategies for Alzheimer’s disease (AD) by modulating the gut‐brain axis.

**Method:**

The present study involved screening a library of 352 human gut microbial metabolites using AD patient‐ induced pluripotent stem cell (iPSC)‐derived neurons for their potential to reduce tau phosphorylation. Among the metabolites associated with decreased tau phosphorylation, we selected the most promising candidates for further validation. Multiple AD models, including AD patient iPSC‐derived neurons, 5XFAD transgenic mouse model, and brain organoids, were utilized to assess the therapeutic effects of these metabolites. The role of Agmatine in alleviating AD pathology was specifically examined, and the underlying mechanism was explored using RNA sequencing in AD iPSC‐derived neurons and 16S rRNA sequencing of fecal samples from 5XFAD mice.

**Result:**

In the primary screening of AD patient iPSC‐derived neurons (*n* = 2), 9 out of 352 (2.6%) and 8 out of 352 (2.3%) gut microbial metabolites were identified as significantly reducing pTau231/total tau levels, respectively (Z score < ‐1.5). Based on high‐throughput screening data, Menaquinone‐4, 4‐Methylcatechol, and Agmatine were selected for further validation. Menaquinone‐4 and 4‐Methylcatechol showed reduced tauopathy in the concentration range of 0.01–10 µM without inducing cytotoxicity. Notably, Agmatine demonstrated substantial reductions in AD‐associated phenotypes across various AD models, without evidence of cytotoxicity. RNA sequencing of Agmatine‐treated AD iPSC‐derived neurons identified the complement system as a key pathway in its mechanism of action. Additional validation in AD transgenic mouse models confirmed the reduced pathology associated with these metabolites.

**Conclusion:**

This study demonstrates successful application of AD patient iPSC‐derived neurons in a phenotypic screening platform to identify gut microbial metabolites capable of reducing pathological tau accumulation in familial AD neurons. These findings provide evidence supporting the therapeutic potential of targeting the human gut‐brain axis to modulate AD pathology, offering a novel therapeutic approach for AD.

